# Risk factor obesity: treatment standard

**DOI:** 10.1093/ndt/gfag164

**Published:** 2026-07-14

**Authors:** Mahtab Mashayekhi, Jianbo Qing, Giv Heidari-Bateni, Roy O Mathew, Adrian Liew, Andreas Kronbichler, Edgar Lerma, Amir Abdipour, Kevin Codorniz, Junnan Wu, Sayna Norouzi

**Affiliations:** Division of Nephrology, Department of Medicine, Loma Linda University Medical Center, Loma Linda, USA; Department of Nephrology, Sir Run Run Shaw Hospital, Zhejiang University School of Medicine, Hangzhou, China; Loma Linda University, School of Medicine, Loma Linda, USA; Division of Nephrology, Department of Medicine, Loma Linda VA Healthcare System, Loma Linda, USA; Mount Elizabeth Novena Hospital, Singapore; Department of Internal Medicine IV, Nephrology and Hypertension, Medical University Innsbruck, Innsbruck, Austria; Division of Nephrology, Department of Medicine, Advocate Christ Medical Center, University of Illinois at Chicago, Oak Lawn, USA; Division of Nephrology, Department of Medicine, Loma Linda University Medical Center, Loma Linda, USA; Diabetes Treatment Center, Loma Linda University, Loma Linda, USA; Department of Nephrology, Sir Run Run Shaw Hospital, Zhejiang University School of Medicine, Hangzhou, China; Division of Nephrology, Department of Medicine, Loma Linda University Medical Center, Loma Linda, USA

**Keywords:** GLP-1RA, gut microbiota, kidney disease, obesity

## Abstract

Obesity is a global epidemic and is increasingly recognized as a direct and independent risk factor for chronic kidney disease (CKD), beyond its established associations with diabetes and hypertension. Excess adiposity promotes kidney injury through convergent hemodynamic, metabolic, and inflammatory pathways, including glomerular hyperfiltration, podocyte stress, lipotoxicity, insulin resistance, activation of the renin–angiotensin system, and chronic low-grade inflammation. Given the rise in the prevalence of obesity and kidney disease driven by obesity as a primary risk factor, there is a critical need to understand emerging treatment options, including novel incretin-based therapies, to prevent disease progression and decrease cardiovascular burden, which is the leading cause of morbidity and mortality in these patients. We aim to review current treatment standards and discuss novel developments in the pathophysiology, diagnosis, outcome prediction, and management of obesity as a risk factor for kidney injury and CKD.

## INTRODUCTION

Obesity is a worldwide epidemic and is currently recognized not only as a cardiovascular and metabolic risk factor, but also as a direct risk factor for kidney injury. Individuals with elevated body mass index (BMI), particularly BMI ≥ 30 kg/m^2^ (≥27.5 kg/m^2^ in individuals of Asian descent), show increased risk for albuminuria, reduced glomerular filtration rate (GFR), and chronic kidney disease (CKD) independent of hypertension and type 2 diabetes (T2D) [1,2]. Histologically, obesity can cause glomerulomegaly, podocyte hypertrophy, and, frequently, perihilar focal segmental glomerulosclerosis, and clinically, may present insidiously with microalbuminuria or subnephrotic proteinuria, which can progress to kidney failure [1,3]. The pathophysiology of kidney injury in obesity demonstrates hemodynamic, metabolic, and inflammatory stresses. Obesity increases kidney plasma flow, estimated glomerular filtration rate (eGFR), and filtration fraction (hyperfiltration), resulting in elevated intraglomerular pressure, glomerular enlargement, and podocyte stress. It can also cause lipotoxicity from ectopic lipid deposition and insulin resistance [3,4]. Glomerular hyperfiltration may represent a central unifying mechanism linking obesity-associated hemodynamic, metabolic, and inflammatory pathways to the progression of kidney injury. Increased proximal tubular sodium reabsorption, altered tubuloglomerular feedback, activation of the renin–angiotensin system (RAS) and sympathetic nervous systems, afferent arteriolar vasodilation, and increased intraglomerular pressure contribute to maladaptive hyperfiltration in obesity-related kidney injury. Sustained glomerular hyperfiltration induces mechanical stress on the filtration barrier, promoting podocyte hypertrophy and loss, mesangial expansion, albuminuria, glomerulosclerosis, tubular overload, and progressive CKD [5–7].

Obesity is a central upstream driver within the cardiovascular–kidney–metabolic continuum, initiating metabolic and cardiovascular abnormalities that contribute to albuminuria, eGFR reduction, and increased cardiovascular risk, collectively termed metabolic-associated kidney disease [8].

Timely diagnosis and management of kidney injury in the setting of obesity are important to prevent irreversible kidney damage and progression to kidney failure. Moreover, obesity management can drive meaningful cardiovascular benefit, because the same obesity-driven pathways that promote glomerular hyperfiltration and proteinuria also accelerate atherosclerosis, heart failure, and cardiometabolic risk. Intentional weight loss has been associated with remission or substantial improvement in proteinuria, supporting the concept that targeting obesity can reverse key hemodynamic and inflammatory drivers of disease. Specifically, interventions such as incretin-based therapies and bariatric surgery have been associated with greater reductions in cardiovascular risk and beneficial kidney outcomes [4].

Here, we review current treatment standards and highlight emerging advances in the pathophysiology, diagnosis, outcome prediction, and management of obesity with kidney injury, informed by a structured literature search in PubMed and Scopus, focusing on studies of obesity-associated kidney injury and relevant therapeutic interventions.

## TREATMENT STANDARDS: LIFESTYLE AND WEIGHT MANAGEMENT: DIET, EXERCISE, AND BEHAVIORAL INTERVENTIONS

Lifestyle modifications and weight management are the cornerstone of obesity treatment. Effective weight management in kidney disease should be delivered within a stigma-aware approach that prioritizes empathetic, patient-centered communication, uses nonstigmatizing language, and addresses prior experiences of weight bias, discrimination, and psychosocial barriers to enhance engagement and clinical outcomes. Sustained weight reduction has been associated with decreased proteinuria, improved glomerular hemodynamics, and stabilization of kidney function in individuals with elevated BMI [4,9,10]. In a study of 63 adults with kidney biopsy findings suggestive of obesity-related glomerulopathy (ORG) in a physician-supervised diet-and-exercise program for 24 months, those who lost weight (about 8%–9% BMI reduction) experienced a 35.3% reduction in proteinuria at 6 months and a 51.3% reduction at 24 months [11].

Lifestyle modifications and weight-management interventions, integrating diet quality, regular physical activity (≥150 min per week of aerobic physical activity), and behavioral counseling, consistently improve cardiometabolic risk factors that are tightly linked to kidney disease progression [12,13]. Intensive, multicomponent programs achieved sustained weight loss, reductions in visceral adiposity, and improvements in insulin sensitivity, lipid profiles, and blood pressure (BP), which are closely linked to kidney disease risk and progression, particularly in populations with obesity, diabetes, and early CKD ( Fig. 2) [12]. Dietary interventions emphasizing Mediterranean or plant-forward approaches were associated with lower prevalence of metabolic syndrome, improved lipid and glycemic control, and reduced mortality, mechanisms that indirectly slow CKD progression through reduced inflammation and oxidative stress [12].

**Figure 1:**
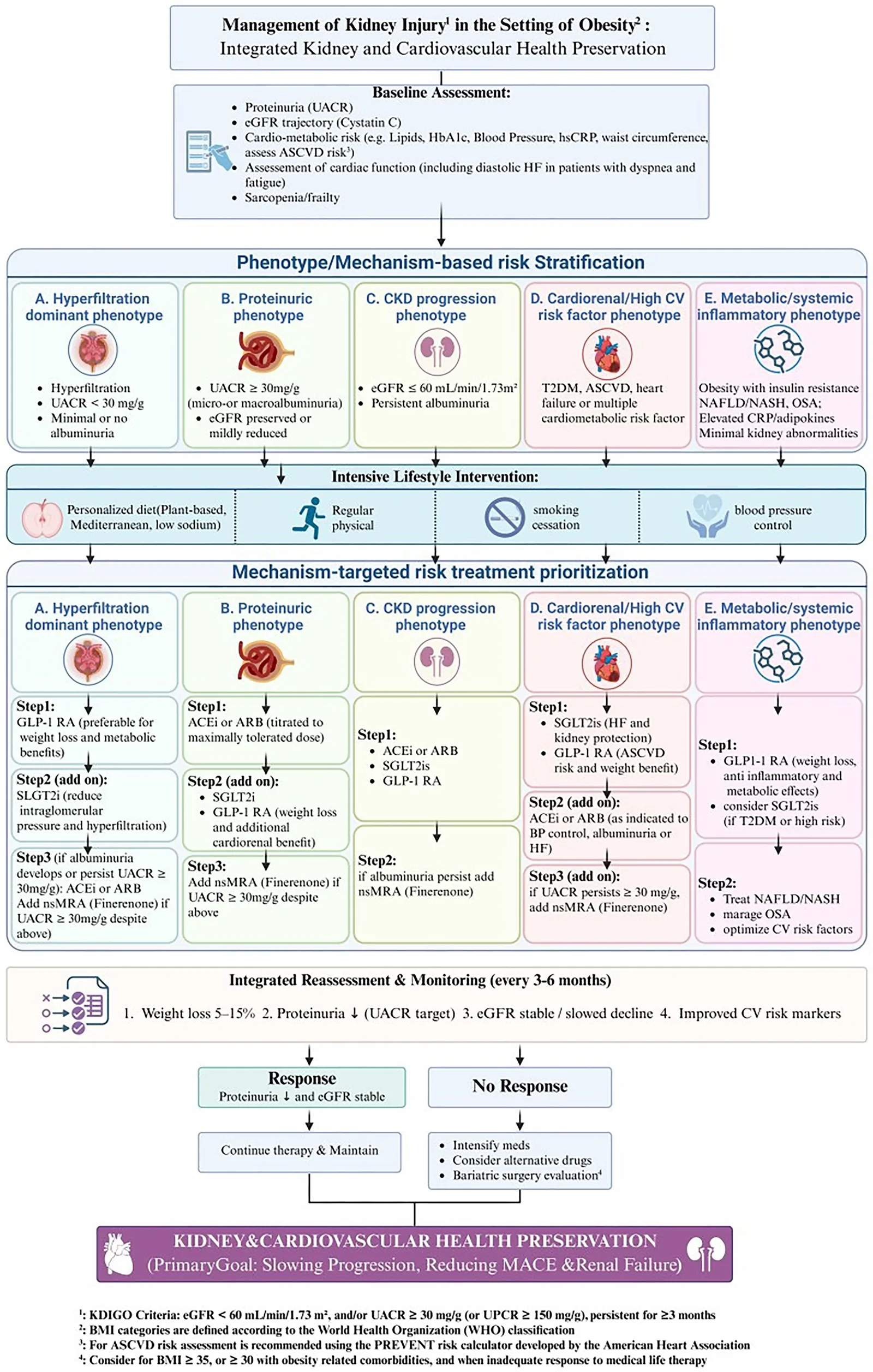
Treatment algorithm. ASCVD, atherosclerotic cardiovascular disease; CV, cardiovascular; HbA1c, glycated hemoglobin; HF, heart failure; hsCRP, high-sensitivity C-reactive protein; NAFLD, nonalcoholic fatty liver disease; NASH, nonalcoholic steatohepatitis; nsMRA, nonsteroidal mineralocorticoid receptor antagonist; T2DM, type 2 diabetes mellitus; UPCR, urine protein-to-creatinine ratio.

**Figure 2:**
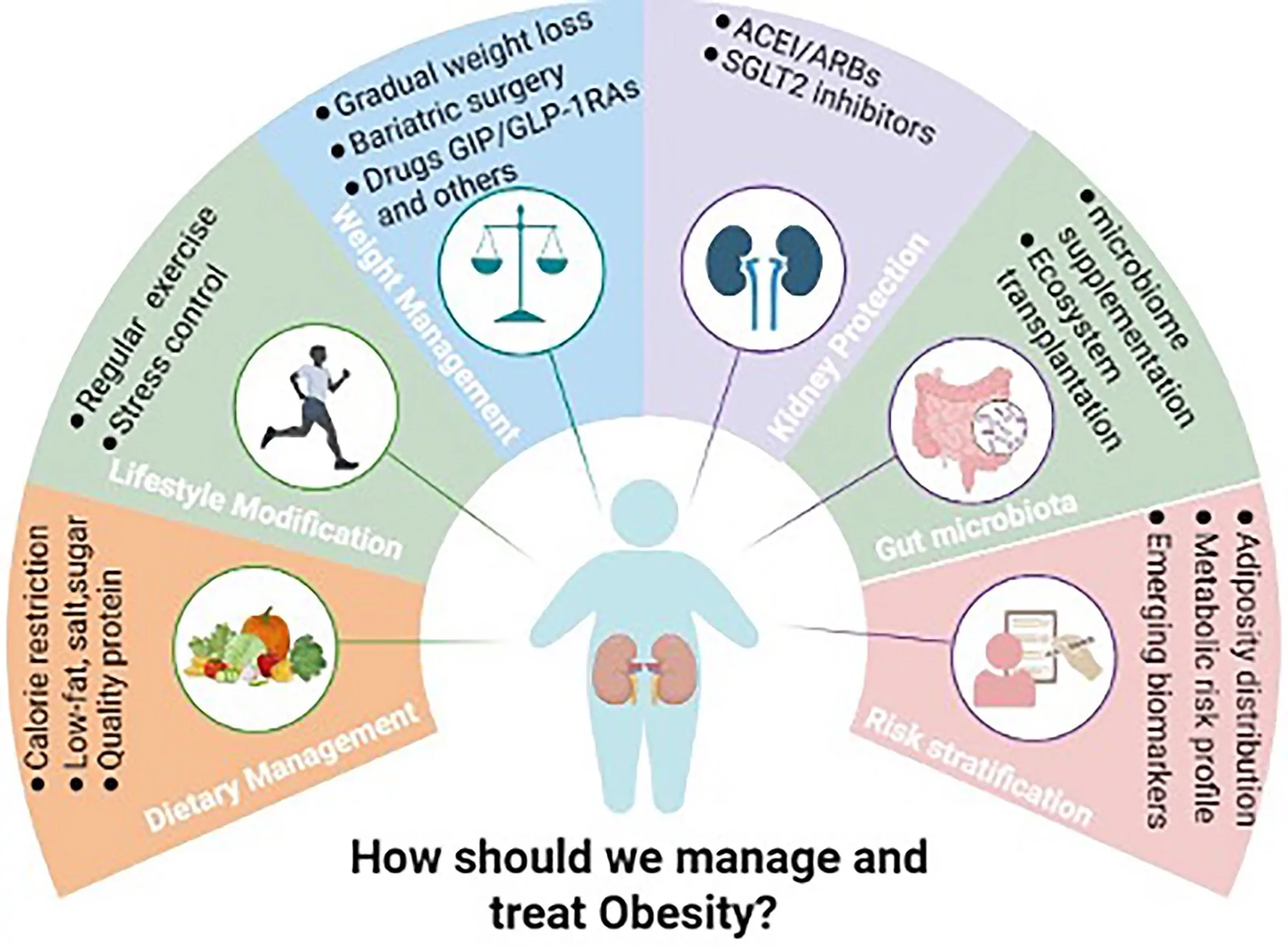
How should we manage and treat obesity.

In the DiRECT 5-year extension study, a structured, diet-based weight management intervention achieved sustained clinically meaningful weight loss; participants in the extended support group maintained an average weight reduction of 6.1 kg at 5 years. Sustained weight loss was associated with prolonged periods of improved metabolic control, reduced reliance on glucose-lowering and antihypertensive medications, and fewer serious adverse events compared with usual care [14].

Sleep duration and quality are also important components of lifestyle modification in obesity and CKD. Because, short sleep duration and circadian disruption are associated with increased weight gain and obesity, changes in appetite regulation and food preferences, and alterations in leptin, ghrelin, insulin, and gut-derived peptide signaling [15]. Addressing sleep hygiene and screening for sleep disorders may therefore represent an important strategy for the management of obesity and its associated cardio-kidney risk.

Obstructive sleep apnea (OSA), which is highly prevalent in obesity, is associated with nocturnal hypertension and nondipping BP patterns, both linked to increased cardiovascular and kidney risk. Intermittent hypoxia and sympathetic and RAS activation in OSA may contribute to endothelial dysfunction, glomerular hyperfiltration, and albuminuria [16]. Therefore, screening for OSA and recognition of nocturnal hypertension may be important components of risk assessment and management in obesity-related kidney injury.

## PHARMACOTHERAPY FOR OBESITY

Modern incretin-based anti-obesity pharmacotherapies, including liraglutide and semaglutide [glucagon-like peptide-1 receptor agonists (GLP-1RAs)] and tirzepatide [dual glucose-dependent insulinotropic polypeptide (GIP)/GLP-1RAs], target weight and cardiometabolic risk at scale, and increasing trial evidence suggests clinically meaningful kidney protection [17]. In the FLOW trial, among 3533 patients with T2D (with mean BMI 32.0 ± 6.3 kg/m^2^) followed for a median of 3.4 years, once weekly subcutaneous semaglutide (1.0 mg) lowered the risk of the primary composite kidney outcome, kidney failure, sustained ≥50% decline in eGFR, or death from kidney-related or cardiovascular causes, by 24% compared with placebo [hazard ratio (HR) 0.76; 95% confidence interval (CI), 0.66–0.88]. Semaglutide also slowed annual eGFR decline by 1.16 ml/min/1.73 m^2^, reduced major cardiovascular events by 18% (HR 0.82), decreased cardiovascular mortality by 29% (HR 0.71), and reduced all-cause mortality by 20% (HR 0.80). Albuminuria in the semaglutide group was reduced, with a 40% reduction in urinary albumin-to-creatinine ratio (UACR) at 104 weeks, compared with a 12% reduction with placebo, corresponding to a 32% lower UACR relative to placebo (UACR ratio 0.68; 95% CI, 0.62–0.75) [18]. In the SELECT trial, once weekly subcutaneous semaglutide 2.4 mg in 17 604 adults with overweight or obesity and established cardiovascular disease without diabetes, reduced the incidence of the composite kidney endpoint, death from kidney disease, initiation of dialysis, persistent eGFR <15 ml/min/1.73 m^2^, sustained ≥50% decline in eGFR, or onset of persistent macroalbuminuria, to 1.8% compared with 2.2% with placebo (HR 0.78; 95% CI 0.63–0.96; *P* = .02). Semaglutide was associated with a slower decline in kidney function, with a difference in mean eGFR change of 0.75 ml/min/1.73 m^2^ at 104 weeks in the overall population, and a larger difference of 2.19 ml/min/1.73 m^2^ among participants with baseline eGFR <60 ml/min/1.73 m^2^. Moreover, semaglutide significantly decreased albuminuria, with a net 10.7% reduction in UACR at 104 weeks, and greater reductions in those with baseline albuminuria [19]. Semaglutide received Food and Drug Administration (FDA) approval for reducing the risk of sustained eGFR decline, end-stage kidney disease, and cardiovascular death in adults with type 2 diabetes mellitus and CKD. Beyond weight reduction, GLP-1RAs exert direct kidney-protective effects through multiple mechanisms. These include promotion of natriuresis via inhibition of proximal tubular sodium–hydrogen exchanger activity, reduction in intraglomerular pressure and hyperfiltration, and modulation of inflammatory and fibrotic pathways within the kidney [20].

In December 2025, the FDA approved the first oral GLP-1RA, semaglutide, for weight loss and to reduce the risk of major adverse cardiovascular events (MACE). In the OASIS-4 randomized, double-blind trial involving adults with overweight or obesity without diabetes, oral semaglutide 25 mg once daily produced substantial weight loss over 64 weeks, with a mean reduction of 13.6% compared with 2.2% with placebo (between-group difference −11.4 percentage points; *P* < .001), and significantly improved physical function and multiple cardiometabolic risk factors, including glycemia, lipids, and inflammatory markers [21].

In a post hoc analysis of the phase 3 SURMOUNT-1 and SURMOUNT-2 trials, tirzepatide treatment was associated with significant improvements in kidney biomarkers in adults with overweight or obesity, with or without T2D. In 3477 participants followed for 72 weeks, tirzepatide reduced UACR compared with placebo, with a placebo-corrected reduction of 28.4% (95% CI, −37.4 to −18.1) in SURMOUNT-1 (obesity without diabetes) and 31.1% (95% CI, −40.9 to −19.7) in SURMOUNT-2 (obesity with diabetes). Albuminuria reduction was substantially greater among participants with baseline UACR ≥30 mg/g, reaching relative reductions of 42.3% and 55.2% in SURMOUNT-1 and SURMOUNT-2, respectively [22].

Despite the substantial efficacy of modern incretin-based therapies, important real-world barriers limit their widespread implementation. High medication costs, inconsistent insurance coverage, and intermittent supply shortages have restricted access to GLP-1RAs and dual incretin therapies in many healthcare settings.

Sympathomimetic anti-obesity agents, such as phentermine and related compounds, reduce weight by activating central noradrenergic pathways but have not demonstrated kidney-protective benefits. In contrast, their pharmacologic stimulation of the sympathetic nervous system may increase heart rate, BP, and renal vasoconstriction, potentially exacerbating glomerular hyperfiltration, intraglomerular hypertension, and cardiovascular risk, particularly in patients with CKD [23].

Orlistat is a gastrointestinal lipase inhibitor used to treat obesity that promotes weight loss by reducing dietary fat absorption in the intestine and is approved for weight management. Although orlistat is effective for modest weight loss, clinical evidence does not demonstrate kidney benefits; rather, multiple case reports and observational series demonstrated that orlistat increases the risk of acute kidney injury and oxalate nephropathy, and population safety analyses suggest an elevated risk of nephrolithiasis and CKD with its use [24].

Naltrexone–bupropion is another approved pharmacologic therapy for obesity that promotes weight loss and improves metabolic risk factors, including reductions in body weight, waist circumference, insulin resistance, and selected cardiometabolic parameters [25]. Through these metabolic effects, it may indirectly reduce the risk of obesity-related kidney disease; however, evidence for kidney-specific effects remains limited, as kidney outcomes such as albuminuria and eGFR have not been systematically evaluated in clinical trials.

A retrospective cohort study of adults with obesity treated with GLP-1 analogues evaluated whether adding bupropion/naltrexone provided incremental weight loss compared with GLP-1 monotherapy. Results showed that GLP-1 analogue monotherapy (liraglutide or semaglutide) was associated with a mean weight loss of 11.4 kg, corresponding to 9.6% total body weight loss (TBWL) at 12 months. In patients who received add-on bupropion/naltrexone after GLP-1 initiation, overall weight loss was not significantly different from monotherapy when analyzed as a single group. However, stratified analyses demonstrated that adding bupropion/naltrexone was associated with additional weight loss of 4.3% TBWL at 6 months and 5.3% at 12 months in patients who had already achieved ≥5% TBWL on GLP-1 therapy, and 3.7% at 6 months and 4.0% at 12 months in initial GLP-1 nonresponders. These findings show that combination therapy may provide clinically meaningful additional weight loss, particularly in individuals with suboptimal early response to GLP-1 agonists [26].

Sarcopenic obesity and frailty are major safety concerns in patients with CKD undergoing weight loss. Excess body weight does not indicate preserved muscle mass, and individuals with kidney injury are particularly vulnerable to muscle loss, functional decline, and frailty during rapid or aggressive weight reduction. Weight-loss strategies in CKD must incorporate resistance exercise, ensure adequate protein intake, and favor slower, individualized weight-loss targets to avoid deterioration of nutritional status and physical function [10]. This is an important issue, because weight loss with GLP-1RAs is accompanied by reductions in both lean and fat mass, and reductions in lean mass may account for a substantial proportion of total weight loss [27]. Data specifically evaluating body composition changes with GLP-1RAs in CKD populations remain limited; however, emerging evidence suggests that muscle quality and metabolic function may improve despite reductions in lean mass [28]. Accordingly, careful monitoring of nutritional status and body composition should be considered during treatment.

Weight regain is common after discontinuing weight-loss interventions, including lifestyle modification and pharmacologic therapy [29]. Therefore, there is an urgent need for sustained lifestyle measures and long-term strategies to achieve durable weight control.

## SGLT2 INHIBITORS AND RAS INHIBITION: AS FIRST LINE FOR PROTEINURIA.

Sodium–glucose cotransporter-2 inhibitors (SGLT2i) play an important adjunctive role in patients with obesity, particularly when metabolic or kidney comorbidities are present. They are producing modest but sustained weight loss primarily through urinary glucose excretion and associated caloric loss [30]. In individuals with obesity, including those without diabetes, SGLT2i typically induce a placebo-adjusted weight reduction of ∼1.5–2 kg, with greater effects observed when combined with appetite-suppressing agents. SGLT2i showed well-established kidney benefits, including reductions in intraglomerular pressure, mitigation of hyperfiltration, and consistent lowering of albuminuria, effects that appear largely independent of glycemic control. Large cardiovascular and kidney outcome trials in patients with diabetes and CKD have demonstrated robust reductions in albuminuria, slowing of eGFR decline, and decreased risk of kidney failure [30]. Empagliflozin demonstrated significant kidney-protective effects across a broad population of patients with CKD, including those with and without diabetes and across a wide range of albuminuria levels. Treatment with empagliflozin caused a modest early decline in eGFR of ∼2.12 (95% CI 1.83 to 2.41) ml/min/1.73 m^2^ within the first two months, followed by a substantial long-term benefit, halving the chronic rate of eGFR decline from −2.75 to −1.37 ml/min/1.73 m^2^ per year (relative reduction 50%, 95% CI 42%–58%). Empagliflozin decreased UACR by 19% (95% CI 15%–23%) compared with placebo [31].

Combination therapy with SGLT2i and GLP-1RAs has emerged as a promising strategy to enhance cardiovascular and kidney protection because these agents target complementary pathophysiologic pathways. Recent reviews and meta-analyses suggest that combined therapy may provide greater cardiorenal benefit than monotherapy alone in patients with obesity, T2D, and CKD [32]. However, additional randomized studies are still needed to better define long-term kidney outcomes and the safety of combination regimens.

RAS inhibitors, including angiotensin-converting enzyme inhibitor (ACEi) and angiotensin receptor blockers (ARBs), have a cornerstone role in the treatment of proteinuric kidney disease in patients with obesity [33], because studies show consistent proteinuria reduction and slower CKD progression in proteinuric CKD, and available obesity-focused analyses suggest these benefits are preserved (and may be increased) in higher-BMI patients. In nondiabetic proteinuric CKD, the REIN trial demonstrated that ACE inhibition with ramipril reduces proteinuria and delays progression to kidney failure, and a post hoc REIN analysis specifically showed strong renoprotection in obese participants [34]. In obesity-related kidney involvement, data support RAS inhibition as an effective antiproteinuric strategy, typically recommended alongside intensive weight management [4,35] (Table 1).

**Table 1:** Pharmacological therapies: doses, markers of response, precautions, and toxicities.

Drug (class)	Typical dose	Markers of response	Key precautions	Common toxicities
**Semaglutide (GLP-1RA)**	0.25 mg weekly SC, titrated to 1.0–2.4 mg weekly, or 25 mg oral tablet once daily	Weight reduction; ↓ albuminuria; CV risk reduction	Caution with severe GI disease; avoid in medullary thyroid carcinoma or MEN2	Nausea, vomiting, diarrhea; gallbladder disease
**Tirzepatide (dual GIP/GLP-1 agonist)**	2.5 mg weekly SC, titrated to 5–15 mg weekly	Marked weight reduction; ↓ albuminuria; improved cardiometabolic risk	Like GLP-1RAs; limited data in advanced CKD	GI intolerance; rare hypoglycemia (with insulin/sulfonylureas)
**Liraglutide (GLP-1RA)**	0.6 mg daily SC, titrated to 1.8–3.0 mg daily	Weight reduction; ↓ HbA1c; ↓ albuminuria; CV benefit	Daily injections; caution in gastroparesis	Nausea, vomiting; injection-site reactions
**Naltrexone–bupropion (central appetite modulator)**	8/90 mg daily, titrated to 16/180 mg twice daily	Modest weight reduction; ↓ waist circumference	Avoid in uncontrolled hypertension, seizure disorders, eating disorders	Nausea, insomnia, anxiety; ↑ BP; seizure risk
**ACEi (e.g. ramipril)**	2.5–10 mg daily (titrated as tolerated)	↓ Proteinuria; ↓ BP; slowed eGFR decline	Check creatinine 7–10 days after initiation; hold during acute illness	Hyperkalemia; cough; AKI (volume depletion, RAS)
**ARB (e.g. losartan)**	50–100 mg daily	↓ Proteinuria; ↓ BP; renoprotection	Avoid combination with ACEi; monitor creatinine	Hyperkalemia; hypotension; AKI
**SGLT2i**				
**Empagliflozin**	10 mg daily	↓ Albuminuria; slowed eGFR decline; HF & CV benefit	Hold during acute illness; caution with recurrent genital infections	Genital mycotic infections; volume depletion; rare euglycemic DKA
**Dapagliflozin**	10 mg daily	↓ Albuminuria; ↓ CKD progression; CV protection	Like empagliflozin; effective down to low eGFR thresholds	Genital infections; hypotension
**Canagliflozin**	100 mg daily	↓ Albuminuria; ↓ kidney failure risk	Monitor volume status; caution with fracture risk	Genital infections; rare amputations (historical signal)

CV, cardiovascular; DKA, diabetic ketoacidosis; GI, gastrointestinal; HbA1c, glycated hemoglobin; HF, heart failure; MEN2, multiple endocrine neoplasia type 2; SC, subcutaneous.

Emerging evidence supports glomerular hyperfiltration reduction as an important therapeutic target in obesity-related kidney disease. RAS inhibitors and SGLT2i may exert complementary renoprotective effects by modulating intraglomerular hemodynamics, including attenuation of intraglomerular hypertension and restoration of tubuloglomerular feedback, ultimately reducing podocyte stress and progressive glomerular injury [5,7].

Additional antiproteinuric agents may have a future. Nonsteroidal mineralocorticoid receptor antagonists such as finerenone have demonstrated reductions in albuminuria and kidney disease progression in proteinuric CKD and may be particularly relevant in obesity-associated metabolic kidney disease because of their anti-inflammatory and antifibrotic effects [36,37]. Similarly, inhibition of the endothelin pathway has emerged as a potential strategy to reduce glomerular hypertension and proteinuria [37,38], but evidence specific to kidney injury related to obesity remains limited.

## BARIATRIC/METABOLIC SURGERY

Metabolic and bariatric surgery (MBS) is considered the most effective durable intervention for severe obesity and obesity-related comorbidities. The 2022 American Society of Metabolic and Bariatric Surgery and International Federation for the Surgery of Obesity and Metabolic Disorders recommend MBS for individuals with BMI ≥35 kg/m^2^ regardless of comorbidity severity, recommend it for BMI ≥30 kg/m^2^ with T2D, and advise it be considered for BMI 30–34.9 kg/m^2^ when substantial or durable weight loss (or comorbidity improvement) is not achieved with nonsurgical therapy [39]. In a large retrospective study including 4322 patients with obesity who underwent MBS and 30 919 nonsurgical controls, followed for a mean of 6.1 years, MBS was associated with a 53% lower risk of the primary kidney composite outcome in participants with T2D (HR 0.47, 95% CI 0.42–0.52) and a 48% lower risk in those without diabetes (HR 0.52, 95% CI 0.46–0.60), with consistent reductions across secondary and tertiary kidney composites and individual outcomes, including ≥40% eGFR decline and serum creatinine doubling [40]. A systematic review and meta-analysis showed that bariatric surgery is associated with significant improvements in multiple renal parameters among obese patients with impaired kidney function. Across pooled analyses, bariatric surgery caused a significant reduction in glomerular hyperfiltration, with dichotomous data showing a relative risk of 0.46 (95% CI, 0.26–0.82), and continuous analyses demonstrating significant decreases in measured and estimated GFR in hyperfiltrating patients, indicating normalization of filtration. Bariatric surgery was also associated with a substantial reduction in albuminuria and proteinuria, with relative risks of 0.42 for albuminuria and 0.31 for proteinuria [41]. In pooled analyses of 105 individuals with severe obesity undergoing bariatric surgery, measured GFR declined by an average of 14% (from 107 to 92 ml/min/1.73 m^2^), with the extent of decline strongly correlated with presurgical GFR. Changes in GFR were only weakly correlated with weight loss and were more associated with baseline kidney function, age, and changes in systolic BP. GFR-estimating equations showed improved accuracy when deindexed and applied in individuals with reduced kidney function, with combined creatinine–cystatin C equations performing best [42]. A study by McTigue *et al*. showed that Roux-en-Y gastric bypass produces significantly greater and more sustained weight loss than sleeve gastrectomy [43].

Pharmacological therapy and MBS are effective options, with choice guided by disease severity, comorbidities, patient preferences, and readiness for long-term treatment. Pharmacological options, particularly GLP-1RAs and dual incretin therapies, are favored as first line in addition to lifestyle intervention in patients with mild to moderate obesity, earlier-stage kidney injury, given their demonstrated benefits on weight reduction, albuminuria, and cardiovascular risk. In contrast, MBS is generally reserved for individuals with severe or refractory obesity, obesity-related complications, or those requiring significant and durable weight loss to improve kidney outcomes but higher procedural risk and need for lifelong follow-up [8]. However, randomized controlled trials directly comparing bariatric surgery with medical management for specific kidney outcomes remain limited, and much of the current evidence is derived from observational studies and secondary analyses.

## NEW DEVELOPMENTS

### Pathophysiology

Obesity and its metabolic sequelae, including insulin resistance, dyslipidemia, and chronic low-grade inflammation, could create a systemic milieu that accelerates kidney injury through convergent pathways such as endothelial dysfunction, oxidative stress, altered adipokine signaling (with a shift from anti-inflammatory adipokines such as adiponectin and omentin toward pro-inflammatory leptin and tumor necrosis factor-α signaling), and maladaptive immune activation. Beyond conventional strategies targeting weight loss, glycemic control, and BP reduction, emerging evidence supports a broader framework that intervenes upstream of kidney damage by reshaping metabolic signaling networks and inflammatory circuits [44]. Among these, modulation of the gut–kidney axis has gained substantial attention as a potentially actionable target [45–47].

Obesity-associated dysbiosis has been linked to metabolic inflammation, impaired intestinal barrier integrity, and altered production of microbial metabolites that may contribute to kidney injury [48,49].

A defining feature of the gut–kidney axis is that microbial alterations translate into systemic metabolite changes that influence kidney health. Three metabolite classes are particularly relevant: short-chain fatty acids (SCFAs), bile acids, and uremic toxins.

SCFAs such as acetate, propionate, and butyrate [50], contribute to epithelial barrier integrity and immune regulation, whereas obesity-associated dysbiosis may impair these protective pathways and promote metabolic inflammation [51,52]. In addition, microbial modulation of bile acid pools shapes host metabolism through receptors such as farnesoid X receptor (FXR) [53]. Obesity-related microbial shifts often favor altered bile acid signaling, contributing to insulin resistance and hepatic steatosis processes that secondarily increase renal risk [54]. Although not yet a direct renal therapy, bile acid signaling represents a plausible cross-organ pathway linking gut microbes to metabolic kidney vulnerability. More importantly, microbiota-derived uremic toxins, particularly trimethylamine N-oxide (TMAO), indoxyl sulfate (IS), and p-cresyl sulfate (PCS). These metabolites originate from dietary precursors (choline/carnitine for TMAO; tryptophan for IS; tyrosine/phenylalanine for PCS), are processed by gut microbes, and subsequently transformed by hepatic enzymes [55]. Importantly, IS and PCS are protein-bound toxins that accumulate early as kidney function declines and are associated with endothelial dysfunction, oxidative stress, and fibrotic remodeling, providing a concrete mechanistic bridge between dysbiosis and kidney injury [56]. Although modulation of the gut–kidney axis represents a promising therapeutic target in obesity-related kidney injury, its role in routine clinical decision making remains investigational. Further studies are needed to determine whether microbiota-targeted interventions may provide clinically meaningful kidney benefits beyond established guideline-directed therapies.

### Diagnosis

Traditional reliance on BMI inadequately represents obesity-related kidney risk. Contemporary approaches emphasize improved risk stratification beyond BMI by integrating measures of body fat distribution and metabolic health. Waist circumference and waist-to-hip ratio better reflect visceral adiposity, which is more associated with insulin resistance, inflammation, and CKD progression than generalized obesity [57].

Assessment of metabolic risk factors, including insulin resistance, dyslipidemia, hypertension, and nonalcoholic fatty liver disease, provides additional prognostic insight [58]. Imaging-based evaluation of ectopic fat depots is an emerging strategy. Renal sinus fat and other renal fat compartments, quantified by computed tomography or magnetic resonance imaging in recent studies, have been associated with kidney-relevant hemodynamic abnormalities and may represent a meaningful marker of obesity-related kidney injury; however, their incremental value beyond standard clinical risk assessment and their role in routine care need to be further studied [59]. However, their use in routine clinical practice remains limited due to cost, accessibility, and lack of standardization. In contrast, waist circumference and waist-to-hip ratio are simple and widely accessible measure of central adiposity that are strongly associated with cardiometabolic risk and adverse kidney outcomes, making it a practical tool for routine risk stratification [60].

### Outcome prediction

Outcome prediction should identify markers that include both baseline kidney risk and responsiveness to weight-targeted interventions. To date, albuminuria (or proteinuria) and eGFR trajectory (e.g. eGFR slope) are the most clinically available measures for prognostication and monitoring treatment response across CKD [61]. Cystatin C is another sensitive marker of early and subtle kidney dysfunction in obesity, being less influenced by muscle mass than creatinine, although its levels are associated with fat mass and BMI. Cystatin C requires careful interpretation in obesity. Adipose tissue contributes to circulating cystatin C, and serum levels may be elevated in obesity independent of kidney function; accordingly, cystatin C-based eGFR alone can underestimate GFR in patients with severe obesity [62]. In this setting, combined creatinine–cystatin C equations provide a more accurate estimate of kidney function, and the 2024 Kidney Disease: Improving Global Outcomes (KDIGO) guideline specifically notes that in class III obesity, eGFRcr-cys is the most accurate approach [63]. Importantly, despite this obesity-related effect on serum cystatin C, the prognostic associations of cystatin C with adverse kidney and cardiovascular outcomes remain valid in people with obesity [64]. Beyond traditional measures such as albuminuria and eGFR, emerging biomarkers including neutrophil gelatinase-associated lipocalin (NGAL), kidney injury molecule-1 (KIM-1), and galectin-3 (Gal-3) , Klotho, and selected microRNAs show promise for detecting early kidney injury and predicting progression in ORG [65].

In addition, longitudinal assessment of these markers is increasingly recognized as more informative than single time-point measurements, particularly in the context of weight-targeted interventions. Dynamic changes in albuminuria, eGFR slope, and cystatin C may capture early treatment responsiveness and help distinguish reversible functional changes from progressive structural injury. Combining traditional kidney markers with emerging biomarkers may further refine risk stratification by identifying subclinical injury and heterogeneous trajectories of disease progression.

### Management

Emerging triple-hormone receptor agonist, retatrutide, that targets GIP, GLP-1, and glucagon receptors, is designed to have synergistic incretin and metabolic effects beyond those seen with single or dual agonists. In a 48-week phase 2 trial, once weekly retatrutide produced marked, dose-dependent weight loss, with mean reductions of 22.8% and 24.2% of baseline body weight at the 8- and 12-mg doses, respectively [66]. In a post hoc analysis of two randomized, placebo-controlled phase 2 trials involving participants with T2D (*n* = 281) or overweight/obesity without diabetes (*n* = 338), retatrutide treatment was associated with favorable kidney-related effects. In participants with T2D, retatrutide 12 mg reduced UACR by 37% at 36 weeks compared with placebo, while eGFR remained stable. In participants with overweight or obesity, retatrutide 8 mg and 12 mg reduced UACR by 28% and 31.5%, respectively, at 48 weeks, and were associated with increases in eGFR derived from creatinine, cystatin C, and combined equations [67]. However, retatrutide remains an investigational therapy, and dedicated kidney outcome trials and long-term safety data are still needed before routine clinical implementation.

Additionally, targeting the gut microbiota represents a promising strategy to simultaneously address obesity and kidney vulnerability. Dietary fiber enrichment, function-guided microbiome supplementation, and microbial ecosystem transplantation can remodel gut microbial communities toward increased SCFA production, improved intestinal barrier integrity, and reduced endotoxemia [68,69]. These changes are associated with improved insulin sensitivity, modest weight reduction, and attenuation of metabolic inflammation. In parallel, microbiome modulation has been shown to lower circulating uremic toxins such as IS and PCS, thereby alleviating kidney inflammatory stress. Emerging clinical and meta-analytic evidence supports the potential of microbiota-targeted interventions to confer combined metabolic and kidney benefits, highlighting the gut–kidney axis as a tractable target for precision prevention strategies in obesity-associated kidney disease [70]. However, microbiome-targeted therapies remain investigational and are not yet established in routine clinical management.

There is an essential need for dedicated kidney outcome trials in nondiabetic obesity, robust data on the long-term safety of GLP-1RAs in patients with kidney injury, development of biomarker-guided treatment strategies, and microbiome-targeted interventions evaluated using kidney-specific endpoints.

## SUMMARY

Obesity is recognized as a direct and modifiable cause of kidney injury, independent of diabetes and hypertension. Through interconnected hemodynamic, metabolic, and inflammatory pathways, excess adiposity promotes glomerular hyperfiltration, podocyte damage, and progressive CKD. Advances in risk stratification now extend beyond BMI to incorporate visceral adiposity, metabolic health, and emerging biomarkers. Early recognition and personalized intervention (Figure 1) are essential to reduce the growing burden of obesity-related kidney disease. While lifestyle modifications are the cornerstone of therapy, modern pharmacologic agents such as incretin-based therapies, SGLT2i, and RAS inhibitors play an important therapeutic role. Bariatric surgery shows durable benefit in selected patients, and emerging treatments such as triple-hormone receptor agonists and modulation of the gut–kidney axis hold promise for future precision strategies. Therapeutic strategies in obesity-related kidney injury may target distinct but overlapping pathophysiologic pathways. SGLT2i and RAS inhibitors primarily exert renoprotective effects through attenuation of maladaptive hyperfiltration and intraglomerular hypertension. GLP-1RAs (in addition to their kidney protective effects) and other anti-obesity therapies predominantly reduce metabolic load, adiposity, insulin resistance, systemic inflammation, and weight-associated hemodynamic stress. Because multiple mechanisms frequently coexist in obesity-related kidney injury, combination approaches targeting both hyperfiltration-driven and metabolic-driven pathways may provide complementary kidney and cardiovascular protection.

Box 1. **“In a nutshell”**

- Obesity is an independent risk factor for kidney injury, causing hyperfiltration, lipotoxicity, RAS activation, and inflammation that can result in glomerulopathy.
- Risk stratification is moving beyond BMI, using visceral adiposity measures, metabolic phenotype, and (emerging) ectopic kidney fat imaging.
- Outcome prediction is becoming dynamic, integrating albuminuria and eGFR slope with adjunct markers (e.g. cystatin C) and emerging tubular/inflammatory biomarkers.
- Incretin-based therapies now have favorable kidney outcomes, with semaglutide and tirzepatide improving albuminuria and slowing kidney decline in high-risk populations.
- Next-generation agents are emerging, including triple-agonists (retatrutide) with striking weight loss and early favorable kidney biomarker signals, while kidney-focused trials ongoing.

Box 2. **Strategies for personalizing treatments**

Personalization starts with phenotyping risk and dominant mechanism rather than BMI alone.

- Define kidney-risk phenotype
  - Quantify albuminuria (UACR) and eGFR trajectory; consider cystatin C when creatinine may be misleading (altered body composition/hyperfiltration).
  - Identify primary mechanisms: hyperfiltration, proteinuria, metabolic risk (T2D, insulin resistance, dyslipidemia, and metabolic dysfunction-associated steatotic liver disease), hypertension, or volume excess.
- Match therapy to phenotype
  - Proteinuria/hyperfiltration predominant: prioritize RAS inhibition (ACEi or ARB), sodium restriction, and add SGLT2 inhibitor when indicated; monitor early eGFR dip and potassium.
  - High cardiometabolic burden and/or need for substantial weight loss: prioritize GLP-1RA or dual GIP/GLP-1 (tirzepatide); retatrutide can be considered as an emerging option where available in trials; monitor gastrointestinal tolerability and dehydration risk, as nausea and vomiting may lead to volume depletion and increase the risk of acute kidney injury, particularly during dose escalation.
  - Advanced CKD or frailty, sarcopenia risk: aim for gradual weight loss, preserve protein intake, and education on resistance training; avoid aggressive caloric restriction.
  - Refractory severe obesity or rapid progression: consider metabolic/bariatric surgery.
  - Because obesity-related kidney injury frequently requires combination therapy targeting weight reduction, proteinuria, hypertension, and metabolic dysfunction simultaneously, treatment sequencing should be individualized. In clinical practice, many clinicians initiate RAS inhibition and/or SGLT2 inhibitors first in patients with albuminuria or hyperfiltration, while GLP-1RAs are added sequentially to enhance weight reduction and cardiometabolic benefit. Staggered initiation may improve tolerability and reduce overlapping risks of gastrointestinal adverse effects, dehydration, hypotension, and acute kidney injury, particularly in patients with advanced CKD, frailty, or concomitant diuretic use.
- Use “treat-to-target” monitoring
  - Targets: ≥5%–10% weight loss (minimum), BP control, and meaningful UACR reduction; reassess response at 3–6 months and consider combination therapy (such as GLP-1RAs with bupropion/naltrexone) if targets are not achieved within this timeframe.

## Supplementary Material

gfag164_Supplemental_File

## Data Availability

No new data were generated or analyzed in support of this research.
