# Supplementary material for: Risk factor obesity: treatment standard
Source: Nephrol Dial Transplant. 2026 Jul 14;41(9):1618–28. doi: 10.1093/ndt/gfag164 (PMC13518094; doi:10.1093/ndt/gfag164)
Supplement: Supplementary file 1 [file gfag164_supplemental_file.docx]

**Table 1Supplementary. Key Randomized Controlled Trials and Major Clinical Studies Evaluating Pharmacologic and Bariatric Interventions**

| **Trial** | **Population** | **Intervention** | **Main Findings** |
| --- | --- | --- | --- |
| FLOW^(1)^ | T2D + CKD (n=3533) | Semaglutide 1.0 mg weekly vs placebo | Reduced composite kidney outcome by 24%; reduced UACR and slowed eGFR decline |
| SELECT^(2)^ | Overweight/obesity + established CVD without diabetes (n=17,604) | Semaglutide 2.4 mg weekly vs placebo | Reduced composite kidney endpoint; decreased albuminuria and slowed eGFR decline |
| SURMOUNT-1^(3)^ | Obesity/overweight without diabetes | Tirzepatide vs placebo | Significant weight loss; post hoc analyses showed reductions in UACR |
| SURMOUNT-2^(4)^ | Obesity + T2D | Tirzepatide vs placebo | Improved body weight and albuminuria-related kidney biomarkers |
| EMPA-KIDNEY^(5)^ | CKD with/without diabetes | Empagliflozin vs placebo | Slowed chronic eGFR decline and reduced albuminuria |
| Retatrutide Phase 2 Trial^(6)^ | Obesity/overweight ± T2D | Retatrutide vs placebo | Marked dose-dependent weight loss up to 24.2% at 48 weeks |
| Swedish Obese Subjects (SOS) Study^(7)^ | Severe obesity | Bariatric surgery vs conventional care | Long-term sustained weight loss and improvement in cardiometabolic risk factors |
| MOMS Trial^(8)^ | Patients with type 2 diabetes, obesity, and early diabetic kidney disease | Roux-en-Y gastric bypass vs best medical treatment | RYGB was more effective than best medical treatment for remission of albuminuria and early CKD at 24 months. |

1.	Perkovic V, Tuttle KR, Rossing P, Mahaffey KW, Mann JF, Bakris G, et al. Effects of semaglutide on chronic kidney disease in patients with type 2 diabetes. New England Journal of Medicine. 2024;391(2):109-21.

2.	Colhoun HM, Lingvay I, Brown PM, Deanfield J, Brown-Frandsen K, Kahn SE, et al. Long-term kidney outcomes of semaglutide in obesity and cardiovascular disease in the SELECT trial. Nature medicine. 2024;30(7):2058-66.

3.	Jastreboff AM, Aronne LJ, Ahmad NN, Wharton S, Connery L, Alves B, et al. Tirzepatide once weekly for the treatment of obesity. New England Journal of Medicine. 2022;387(3):205-16.

4.	Garvey WT, Frias JP, Jastreboff AM, le Roux CW, Sattar N, Aizenberg D, et al. Tirzepatide once weekly for the treatment of obesity in people with type 2 diabetes (SURMOUNT-2): a double-blind, randomised, multicentre, placebo-controlled, phase 3 trial. The Lancet. 2023;402(10402):613-26.

5.	Group E-KC. Empagliflozin in patients with chronic kidney disease. New England Journal of Medicine. 2023;388(2):117-27.

6.	Jastreboff AM, Kaplan LM, Frías JP, Wu Q, Du Y, Gurbuz S, et al. Triple–hormone-receptor agonist retatrutide for obesity—a phase 2 trial. New England Journal of Medicine. 2023;389(6):514-26.

7.	Sjöström L, Narbro K, Sjöström CD, Karason K, Larsson B, Wedel H, et al. Effects of bariatric surgery on mortality in Swedish obese subjects. New England journal of medicine. 2007;357(8):741-52.

8.	Cohen RV, Pereira TV, Aboud CM, Petry TBZ, Lopes Correa JL, Schiavon CA, et al. Effect of gastric bypass vs best medical treatment on early-stage chronic kidney disease in patients with type 2 diabetes and obesity: a randomized clinical trial. JAMA surgery. 2020;155(8):e200420.
